# Tumor suppressor ZHX2 inhibits NAFLD–HCC progression via blocking LPL-mediated lipid uptake

**DOI:** 10.1038/s41418-019-0453-z

**Published:** 2019-11-18

**Authors:** Zhuanchang Wu, Hongxin Ma, Liyuan Wang, Xiaojia Song, Jie Zhang, Wen Liu, Yutong Ge, Yang Sun, Xiangguo Yu, Zehua Wang, Jianping Wang, Yankun Zhang, Chunyang Li, Nailin Li, Lifen Gao, Xiaohong Liang, Xuetian Yue, Chunhong Ma

**Affiliations:** 1 Key Laboratory for Experimental Teratology of Ministry of Education and Department of Immunology, School of Basic Medical Science, Shandong University, Jinan, Shandong 250012 PR China; 2 Clinical Laboratory, Shandong Cancer Hospital & Institute Affiliated to Shandong University, Shandong Academy of Medical Sciences, Jinan, Shandong 250012 PR China; 3 Department of General Surgery, Provincial Hospital Affiliated to Shandong University, Jinan, Shandong 250012 PR China; 4 Karolinska Institutet, Department of Medicine-Solna, Clinical Pharmacology Group, 171 76 Stockholm, Sweden; 5 Key Laboratory for Experimental Teratology of Ministry of Education and Department of Cell Biology, School of Basic Medical Science, Shandong University, Jinan, Shandong 250012 PR China

**Keywords:** Cancer metabolism, Tumour-suppressor proteins, Metabolic disorders

## Abstract

Non-alcoholic fatty liver disease (NAFLD) leads to hepatocellular carcinoma (HCC). However, the underlying mechanism remains largely unclear. Here, we investigated the role of the tumor suppressor Zinc fingers and homeoboxes 2 (ZHX2) in the progression of NAFLD to HCC. ZHX2 expression was significantly decreased in fatty liver tissues, especially in the liver with NAFLD–HCC. ZHX2 overexpression disturbed lipid homeostasis of cultured HCC cells, and inhibited lipid deposition in hepatocytes both in vitro and in vivo. Moreover, ZHX2 inhibited uptake of exogenous lipids through transcriptional suppression of lipid lipase (LPL), leading to retarded proliferation of HCC cells. Importantly, LPL overexpression significantly reversed ZHX2-mediated inhibition of HCC cell proliferation, xenograft tumor growth, lipid deposition, and spontaneous liver tumor formation. Consistently, IHC staining demonstrated a negative correlation of ZHX2 with LPL in an HCC cohort. Collectively, ZHX2 protects hepatocytes from abnormal lipid deposition in NAFLD through transcriptional repression of LPL, which subsequently retards cell growth and NAFLD–HCC progression. These findings illustrate a novel mechanism of NAFLD progression into HCC.

## Introduction

Hepatocellular carcinoma (HCC) is the third leading cause of cancer-related death worldwide. Numerous studies have indicated non-alcoholic fatty liver disease (NAFLD) as a key trigger of HCC, to which the underlying mechanism remains elusive [1, 2]. In the past decades, the morbidity of NAFLD has been dramatically increased, due to the marked increase of metabolic syndromes, such as obesity and diabetes [3]. Therefore, there is an urgent need to clarify the mechanisms underlying NAFLD–HCC progression for developing new therapeutic strategies against HCC.

Over the last decades, there have been steady advances in the understanding on the molecular mechanisms of NAFLD–NASH–HCC progression [1, 4]. It has been established that lipid metabolism disorder plays a critical role in NAFLD–HCC progression [5]. Accumulating evidence demonstrated that tumor suppressor genes (TSGs) closely regulate cell proliferation and differentiation, as well as lipid metabolisms and NAFLD. TP53, a TSG and one of the most frequently mutated genes in HCC, inhibits the expression of cellular metabolism-regulating genes to prevent tumor development [6]. Liver-specific deficiency of PTEN, another well-known TSG, promotes hepatic expression of adipocyte-specific genes and lipogenesis and beta-oxidation-related genes [7], leading to steatohepatitis and HCC [8]. All the evidence strongly suggests that TSGs are important links between NAFLD and HCC. Therefore, identifying TSGs involved in the regulation of lipid metabolisms would be beneficial for understanding mechanisms of NAFLD-related carcinogenesis of HCC.

Zinc fingers and homeoboxes 2 (ZHX2), a member of the ZHX family, has been identified as an HCC-associated TSG [9]. Murine studies suggested that ZHX2 is responsible for postnatal repression of HCC-related genes, such as AFP, GPC3, and H19 [10, 11]. ZHX2-mediated suppression of AFP and GPC3 transcription was also confirmed in cultured HCC cells [12, 13]. Clinical and functional data demonstrated that reduced ZHX2 expression in HCC enhanced cell growth and chemo-resistance. Mechanical studies have identified Cyclin A, Cyclin E, and MDR1 as the transcriptional repression targets of ZHX2 [14–16]. Interestingly, recent research identified ZHX2 as a novel regulator of plasma levels of lipids, including TG [17], indicating a potential role of ZHX2 in lipid metabolism. However, the roles of ZHX2 in NAFLD and NAFLD–HCC progression remains unknown.

Here, we found that ZHX2 inhibited uptake of exogenous lipids in hepatocytes and suppressed NAFLD progression by transcriptionally repressing lipid lipase (LPL) expression. The latter caused cell growth retardation, and inhibited progression of NAFLD to HCC. These findings provide potential therapeutic targets for NAFLD-associated HCC.

## Methods and materials

### Cells, transfection, and luciferase assays

The human HCC cell lines HepG2, Huh7, SMMC7721, QSG7701, Bel7402, and Doxycycline (Dox)-induced Bel7402-ZHX2-Teton cell line were used for cell proliferation and/or colony formation assays. Luciferase assays were used for transcriptional regulation analyses.

### Lipidomics analysis

Bel7402-ZHX2-Teton cells were cultured with Dox (1 μg/ml) for 48 h to induce ZHX2 expression. The cells were collected in 1 ml methanol, and thoroughly mixed on vortex for 15 s followed by pelleting the protein precipitate at 140,000 g. Supernatant (100 μl) was stored at −80 °C refrigerator before lipidomics analysis by Beijing Bio-Tech Pack Technology Company Ltd.

### Clinical samples, immunohistochemical (IHC), and immunofluorescence (IF) staining

Clinical study protocol was approved by the Shandong University Medical Ethics Committee. Fresh HCC and paired paracancer tissues were collected from Shandong Provincial Hospital. Informed consent was obtained from all patients. Tissue microarray containing 75 HCC tissues and paired paracancer tissues was purchased from Superchip (Shanghai, China). LPL expression was analyzed in tissue microarray (*n* = 150) by IHC staining or in fresh tissues (*n* = 40) by qRT-PCR. Correlation between ZHX2 and LPL correlation was detected in HCC tissue microarray (*n* = 75) and paraffin-embedded fresh HCC tissues (*n* = 45). Freshly collected paracancer tissues with (fatty liver) or without (non-fatty liver) vacuolation were used to detect ZHX2 expression by IF staining as previously described [18].

### NAFLD, NAFLD–HCC model, and xenograft tumor assays

To generate liver-specific ZHX2 knockout mice, C57BL/6(BL6) mice with exon three flanked by *loxP* sites (gifted by Dr Brett T. Spear from University of Kentucky) [19]. These mice were crossed with BL/6 mice expressing *Cre* recombinase driven by liver-specific albumin promoter (Alb-Cre) (Shanghai Model Organisms Center, Inc., China) to obtain heterozygous for the floxed *Zhx2* allele with Cre recombinase. Further breeding was performed to obtain homozygous for floxed *Zhx2* allele with or without Alb-Cre transgene (designated as ZHX2-KO^hep^ or ZHX2-WT). DNAs were extracted from the mice tail biopsies. Genotyping of *Zhx2* (flox) and *Cre* transgene were performed using primers as previously described [19]. Eight-week-old ZHX2-KO^hep^ (*n* = 6) and ZHX2-WT (*n* = 6) mice were fed with HFD to induce NAFLD.

NAFLD murine models were induced by feeding with MCD diet or HFD. Briefly, 8-week-old mice were fed with MCD diet (MD12052, Medicinece, Jiangsu, China) for 4 weeks. Four-week-old mice were fed with HFD (MD12032, Medicinece, Jiangsu, China) for 12 weeks. Each group contains 6 mice.

NAFLD–HCC was induced as previously described [20]. Briefly, 200 μg STZ (Sigma) was subcutaneously injected into neonatal male mice (*n* = 36) at 2 days after birth. At week 4, mice were intravenously injected with AAV viruses expressing ZHX2 (AAV-ZHX2) or LPL (AAV-LPL) (5 × 10^11^ PFU) and then fed with HFD. At month 5, tumor nodes in murine livers were analyzed by magnetic resource scan.

H22 cell xenograft tumors were prepared in male BALB/c nude mice (6–8 weeks) for tumor growth analyses. Mice (*n* = 24) were divided in four groups (Con, ZHX2, LPL, and ZHX2/LPL), each group contained at least five mice. Tumor size was monitored every 3 days. Mice were sacrificed at day 15, and xenograft tumors were isolated and weighted.

### Statistical analysis

Data are presented as mean ± SEM from triplicate experiments or mean ± SD from at least three samples. Significance was determined using Student’s *t*-test or one-way ANOVA. A *p* < 0.05 was considered as significant difference.

## Results

### Decreased expression of ZHX2 in fatty liver tissues and hepatocytes

**Fig. 1:**
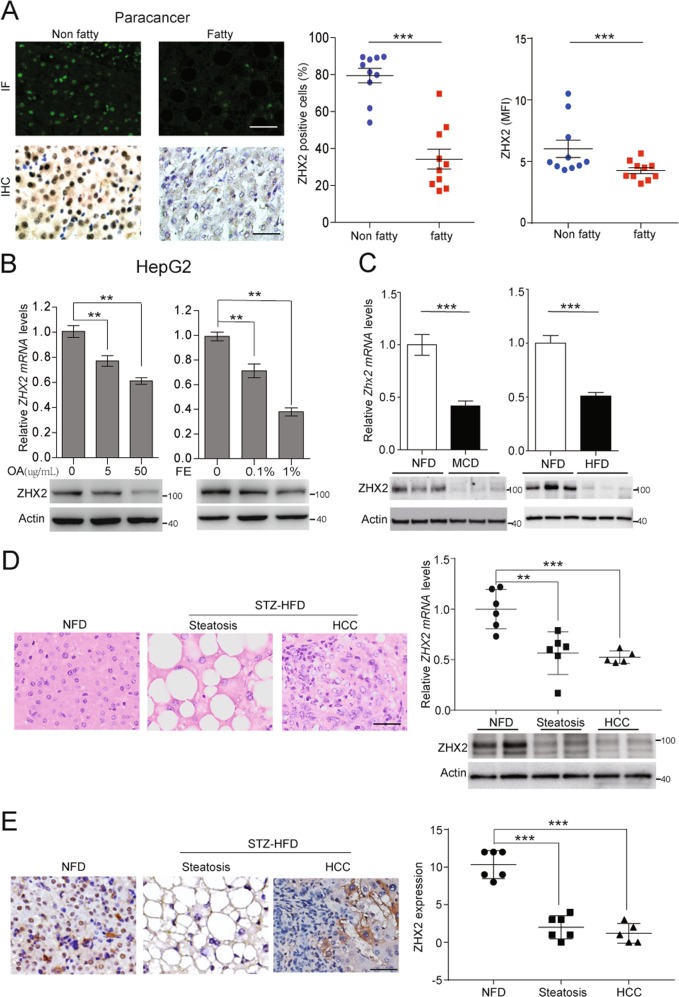
ZHX2 expression was decreased in lipids-treated HepG2 cells and fatty livers. **a** Expression of ZHX2 in human non-fatty liver specimens and fatty liver specimens were determined by IHC and IF. The representative images were shown on the left panel. The right panel showed the percentages of ZHX2 positive cells and florescence intensity of ZHX2 in the specimens. *n* = 10; ***p < 0.001. **b** ZHX2 expression was detected in oleic acid (OA)- or fat emulsion (FE)-treated HepG2 cells by RT-PCR and Western blot. The quantification data were shown on the upper panel. *n* ≥ 6; **p < 0.01. Western blot results were shown on the lower panel. **c** ZHX2 expression was determined in fatty livers from MCD diet- or HFD-induced mice by RT-PCR and Western blot. The quantification of ZHX2 mRNA levels was shown on the upper panels. *n* ≥ 4; ***p < 0.001. ZHX2 protein levels were shown on the lower panel. **d** STZ–HFD was used to induce NAFL**–**HCC murine model. Steatosis and HCC as two stages in the progression of NAFLD to HCC were determined by H&E staining and shown on the left panel. The right panel showed the ZHX2 expression at mRNA and protein levels in liver tissues from the mice fed with non-fatty diet (NFD) and pathological tissues induced by STZ–HFD. **p < 0.01, ***p < 0.001. **e** ZHX2 expression in mice liver tissues was confirmed by IHC staining and showed on left panel. Right panel showed the quantification of ZHX2 IHC staining. ***p *<* 0.001. Data presented are mean ± SEM, *n* = 6

Previous reports demonstrated that ZHX2 expression was decreased in HCC [14, 15]. Interestingly, immunofluorescence (IF) and immunohistochemical (IHC) staining showed that ZHX2 expression significantly decreased in paracancer liver tissues with vacuolation compared with that without vacuolation (Fig. 1a). Liver tissue with vacuolation is a sign of lipid deposition [21], above results indicated that lipids may regulate ZHX2 expression. To address this issue, oleic acid (OA) or fat emulsion (FE) was employed to treat HepG2 cells. As shown in Fig. 1b, ZHX2 expression was clearly decreased on both mRNA and protein levels in OA- and FE-treated HepG2 cells in a dose-dependent manner. Consistent with the in vitro assays, mRNA, and protein levels of ZHX2 were significantly decreased in MCD diet- and HFD-induced fatty livers compared with livers from non-fat diet (NFD) fed mice (Fig. 1c). Furthermore, ZHX2 mRNA and protein levels were significantly decreased in the liver from STZ–HFD induced NAFLD–HCC mice, and the reduction of ZHX2 was clearly observed in both liver steatosis and HCC stage (Fig. 1d, e), indicating the potential involvement of ZHX2 in progress of NAFLD to HCC.

### ZHX2 inhibits hepatic lipid accumulation and NAFLD progression

**Fig. 2:**
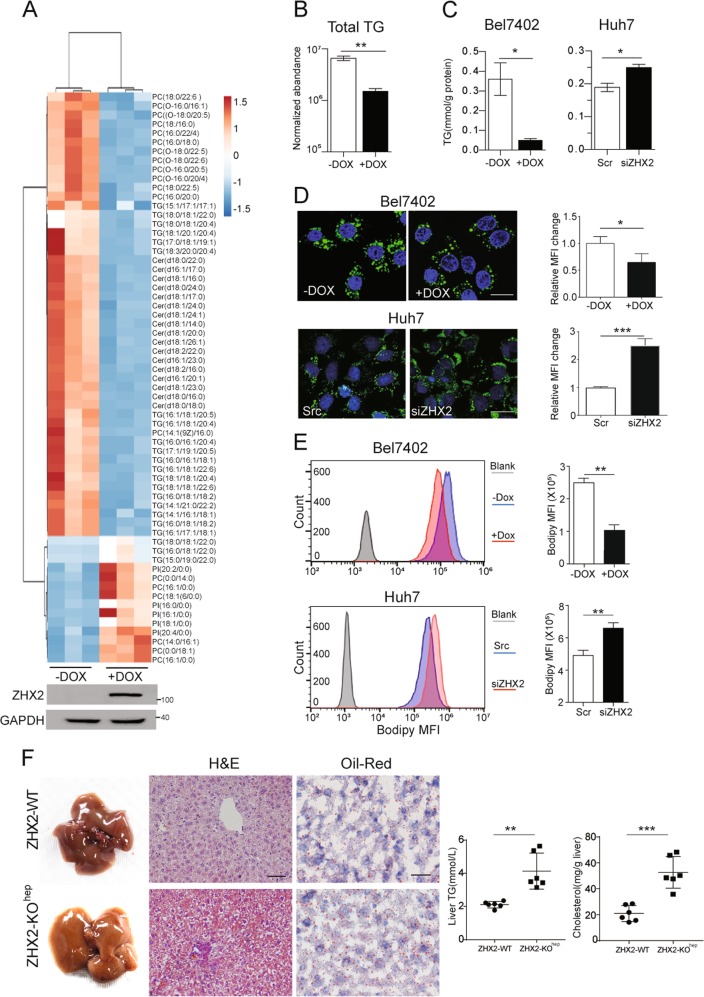
ZHX2 inhibits lipid accumulation in hepatocytes and delays the progression of NFALD. **a** Heat map of lipid metabolites were identified by LC-MS in Bel7402 with or without Dox-induced ZHX2 expression. ZHX2 expression was confirmed by western blot. **b** Normalized abundance of total TG analyzed from lipidomics data of Bel7402 in **a** *n* = 3; **p *<* 0.01. **c**–**e** HCC cell lines with ZHX2 overexpression or knockdown were treated with PA to induce steatosis. Levels of TG (**c**), fluorescence of Bodipy (**d**), and flow cytometry (**e**) were used to determine accumulation of lipids. Statistic results of lipids intensity were shown on the right panel. *n* ≥ 4; *p < 0.05, **p *<* 0.01. **f** The mice with liver-specific knockout of ZHX2 and the control littermates were used to induce NAFLD by feeding with HFD. The representative images of whole mouse liver, Oil Red O, and H&E staining were shown on the left panel. Right panel showed the statistic result of TG and cholesterol levels in liver homogenates. *p < 0.05. Data presented are mean ± SEM, *n* = 6

To clarify the role of ZHX2 in hepatic lipid homeostasis, total lipids of Bel7402 with or without Dox-induced ZHX2 ectopic expression were extracted to perform lipidomics. As shown in Fig. 2a, ZHX2 ectopic expression dramatically decreased most of lipid components, including TG, phosphatidylcholines (PC), and ceramide (Cer). Especially, the normalized abundance of total TG was clearly reduced in Dox-treated Bel7402-ZHX2-Teton cells (Fig. 2b). Accordingly, ectopic expression of ZHX2 clearly inhibited TG accumulation in Bel7402 cells, and knockdown of endogenous ZHX2 markedly increased TG level in Huh7 cells (Fig. 2c). Results of Bodipy staining, which indicates intracellular lipid levels, showed that ZHX2 inhibited lipid accumulation in HCC cell lines (Fig. 2d). These results were further verified by flow cytometry analyses (Fig. 2e) and fluorescence images (Fig. S1A, B). All the results suggest that ZHX2 is an inhibitory regulator in hepatic lipid homeostasis.

To confirm the in vitro findings, Alb-cre was used to delete loxP-flanked *Zhx2* in murine hepatocytes to establish liver-specific ZHX2 knockout mice (ZHX2-KO^hep^) (Fig. S1C). ZHX2-KO^hep^ mice and control littermates (ZHX2-WT) were fed with HFD to induce NAFLD. Hepatic ZHX2 deficiency presented a fatty color for the liver, and increased vacuolation in the liver tissue of ZHX2-KO^hep^ mice, suggesting aggravated liver lipid deposition. A similar trend was also observed by Oil Red O staining (Fig. 2f). Consistently, hepatic levels of total TG and cholesterol were significantly higher in ZHX2-KO^hep^ mice than ZHX2-WT mice (Fig. 2f). In MCD-diet fed mice, knockdown of ZHX2 by lentivirus expressing ZHX2 shRNA significantly increased liver lipid deposition and hepatosteatosis (Fig.S1D). Collectively, our data indicate that ZHX2 inhibits lipid deposition in the liver, and ameliorates NAFLD in mice.

### ZHX2 inhibits HCC cell proliferation by limiting lipid uptake

**Fig. 3:**
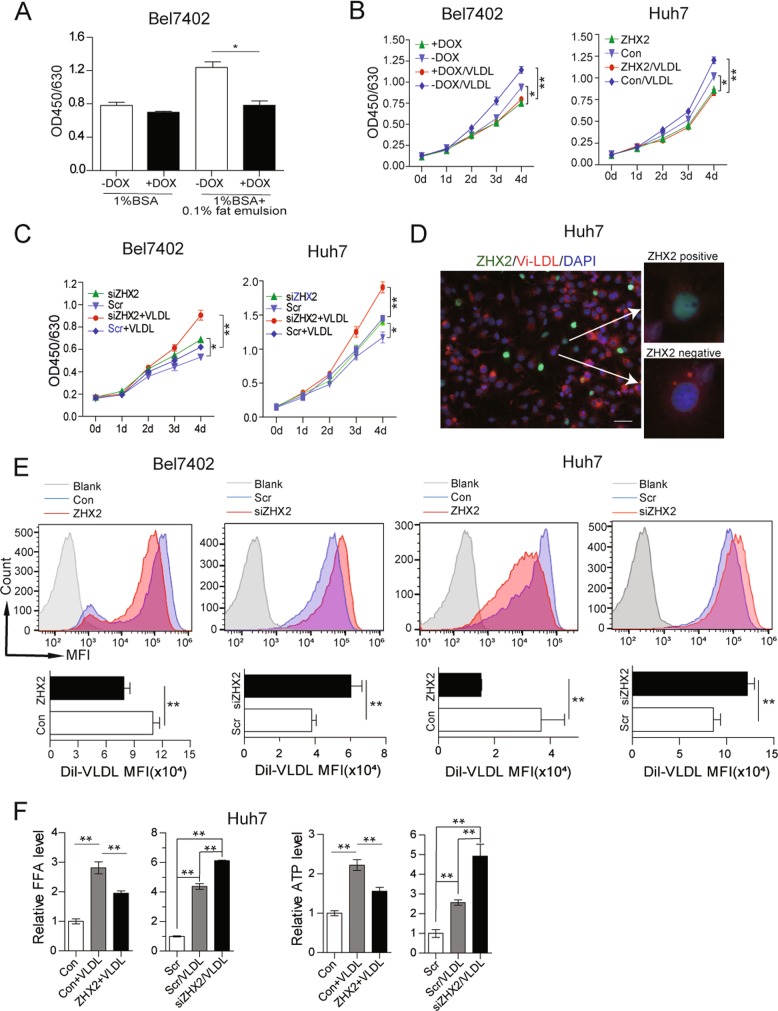
ZHX2 inhibits cell proliferation of HCC cells by blocking lipids uptake. (**a**) Bel7402 cells with or without ZHX2 overexpression were cultured in low glucose medium with 1% fatty acid-free BSA or 1% fatty acid-free BSA plus 0.1% fat emulsion to assess cell proliferation. Bel7402 and Huh7 cells with ZHX2 overexpression (**b**) or knockdown (**c**) were cultured in low glucose medium with or without VLDL. Cell proliferation was assessed by a CCK8 assay kit. **d** Dil-VLDL treated Huh7 cells with overexpression of EGFP-tagged ZHX2. ZHX2 localization and VLDL intensity were shown by the representative images. **e** Bel7402 and Huh7 cells with overexpression or knockdown of ZHX2 were treated with Dil-VLDL. Dil-VLDL intensity was accessed by flow cytometry. **f** Huh7 cells with ZHX2 overexpression or knockdown were treated with VLDL to measure levels of free fatty acids (FFAs) and ATP. *p < 0.05, **p < 0.01. Data presented are mean ± SEM, *n* ≥ 3

A number of recent reports have demonstrated the importance of exogenous lipids in tumor cell proliferation, metastasis and survival [22, 23]. Consistently, HepG2 cell proliferation was decreased in the medium with 1% fatty acid-free BSA compared with that with 10% FBS, and 0.1% FE partially rescued HepG2 cell proliferation (Fig. S2A). To further elucidate the involvement of ZHX2-mediated lipid deposition in its tumor suppressor function, Bel7402 and HepG2 cells were cultured in low glucose medium to minimize *de novo* lipid synthesis. As shown in Fig. S2B and Fig. 3a, ZHX2 overexpression inhibited HCC cell proliferation in low glucose medium with 10% FBS, but the inhibitory effect of ZHX2 was absent when cells were cultured with 1% fatty acid-free BSA. However, the inhibitory effect of ZHX2 re-emerged when supplement with 0.1% FE (Fig. 3a), indicating that ZHX2’s inhibitory effect is partially dependent on exogenous lipids. To verify this finding, Bel7402-ZHX2-Teton and ZHX2-overexpressed Huh7 were cultured in low glucose medium containing VLDL, which can provide exogenous lipids [24]. As shown in Fig. 3b, ZHX2-mediated inhibitory effect on cell proliferation was more obvious in the medium with VLDL than that without VLDL. Reciprocally, ZHX2 knockdown led to more significantly enhanced cell proliferation in Bel7402 and Huh7 cells when cultured in the medium with VLDL than that without VLDL (Fig. 3c). These results suggest that ZHX2 inhibits cell proliferation in an exogenous lipid utilization-dependent manner.

To further validate the role of ZHX2 in limiting uptake of exogenous lipids and inhibiting cell proliferation, Dil-labeled VLDL was employed as the tracker of exogenous lipid uptake in Huh7. Fluorescence images showed that fluorescence intensity of Dil-VLDL was weak in ZHX2-postive cells compared with ZHX2-negative Huh7 cells (Fig. 3d and Fig. S2C). This was further confirmed in Bel7402 and Huh7 cells with manipulation of ZHX2 expression. Both flow cytometry and fluorescence images showed that overexpression of ZHX2 inhibited Dil-VLDL uptake and knockdown of ZHX2 increased Dil-VLDL uptake (Fig. 3e and Fig. S2D). More importantly, VLDL led to increase of free fatty acids (FFAs) and ATP in Huh7 cells, both of which are important resources for cell proliferation. Overexpression of ZHX2 inhibited VLDL-induced increasing of FFAs and ATP, while knockdown of ZHX2 increased VLDL-induced increase of FFAs and ATP in Bel7402 (Fig. 3f). Taken together, all the data suggest that ZHX2 blocks lipid uptake of HCC cells, and therefore inhibits cell proliferation.

### LPL is the direct target of ZHX2

**Fig. 4:**
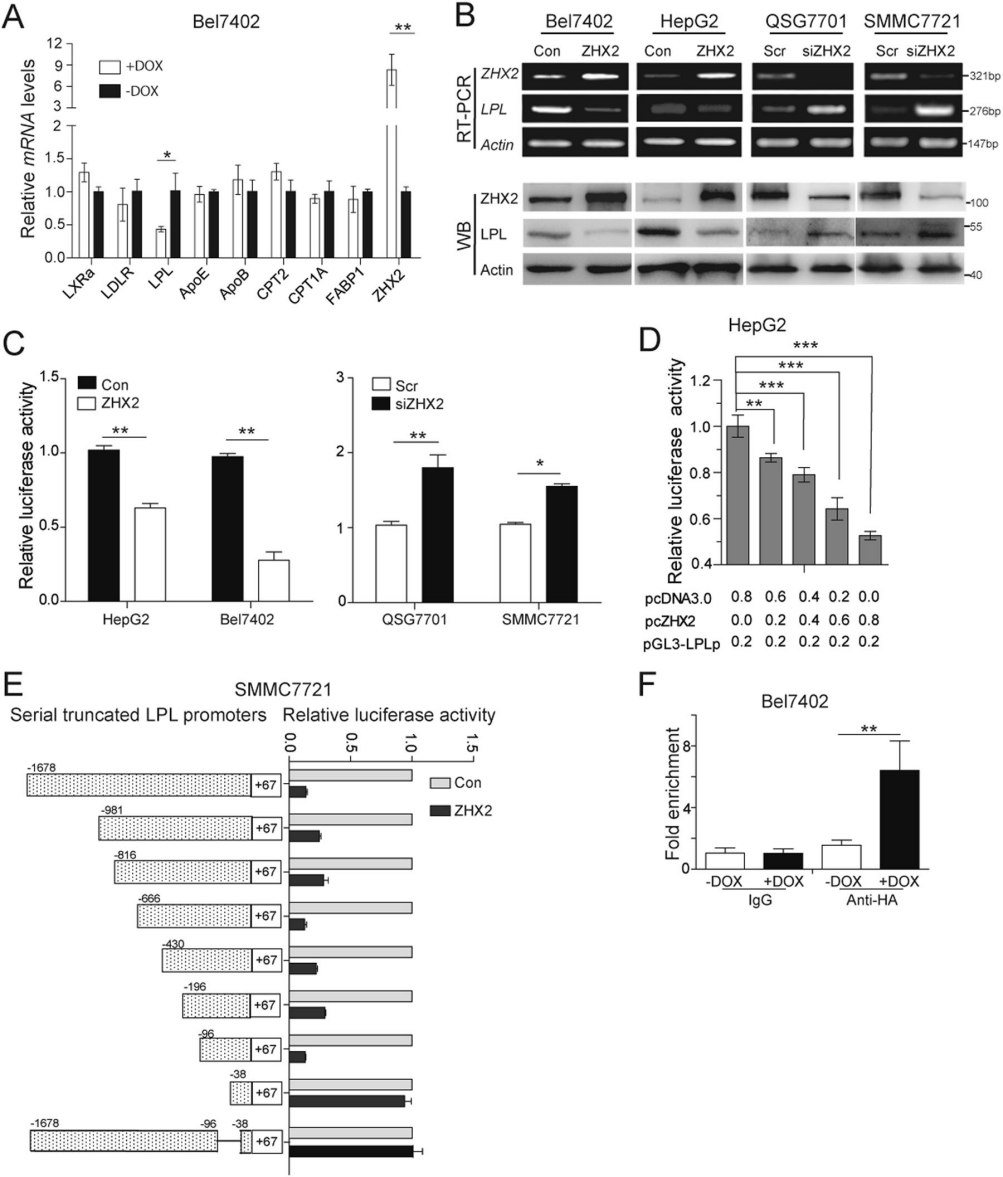
ZHX2 binds to LPL promoter to repress ZHX2 expression at transcriptional level. **a** Lipid homeostasis related genes were screened in Bel7402 cells with or without Dox-induced ZHX2 overexpression by RT-PCR. *n* = 3; *p *<* 0.05, **p *<* 0.01. **b** LPL expression was determined in Bel7402 and HepG2 cells with ZHX2 overexpression or QSG7701 and SMMC7721 cells with ZHX2 knockdown by RT-PCR and Western blot. **c** Luciferase activity of pGL3-LPLp was measured in Bel7402 and HepG2 cells with ZHX2 overexpression or QSG7701 and SMMC7721 cells with ZHX2 knockdown. **d** LPL promoter reporter vector was co-transfected with increased amounts of ZHX2 expression vector in HepG2 cells to measure LPL promoter activity. **e** Series of LPL promoter truncates were constructed, then co-transfected with ZHX2 expression vector in SMMC7721 for luciferase assays. **f** The genomic DNA fragments were pulled down from Bel7402-ZHX2-teton cells by using anti-HA antibody, then used the specific primers targeting −96∼ + 67nt to do qPCR. *n* = 4; *p *<* 0.05, **p *<* 0.01

To identify the potential ZHX2 targets that regulate hepatic lipid homeostasis, RT-PCR was used to screen well-known lipid homeostasis related genes. As shown in Fig. 4a, LPL expression was clearly decreased in Bel7402-ZHX2-Teton cells. This result was confirmed in Bel7402, HepG2, QSG7701, and SMMC7721 cells with ZHX2 overexpression or knockdown (Fig. 4b). As ZHX2 is a transcriptional regulator [9], we hypothesized that ZHX2 might represses LPL expression at transcriptional level. To test this hypothesis, luciferase reporter assays were performed. Ectopic expression of ZHX2 significantly repressed activity of LPL promoter (–1678 to +67nt), and knockdown of endogenous ZHX2 clearly activated LPL promoter (Fig. 4c). Of note, ZHX2 suppressed LPL promoter activity in a dose-dependent manner (Fig. 4d).

To identify the core region of LPL promoter responded to ZHX2, a series of truncated LPL promoter reporter plasmids were constructed. Transient co-transfection followed by luciferase reporter assay showed that truncated LPL promoter retained responsive to ZHX2 until the −96 to −38nt were deleted, suggesting that ZHX2 transcriptionally regulated LPL via region of −96 to −38nt in its promoter (Fig. 4e). Moreover, ChIP assay further confirmed the recruitment of ZHX2 in LPL promoter. As shown in Fig. 4f, ZHX2 dramatically enriched in the region (−96 to +67nt) of LPL promoter when Bel7402-ZHX2-Teton were induced by Dox. Thus, ZHX2 transcriptionally represses LPL expression by binding, directly or indirectly, to the promoter region of LPL.

**Fig. 5:**
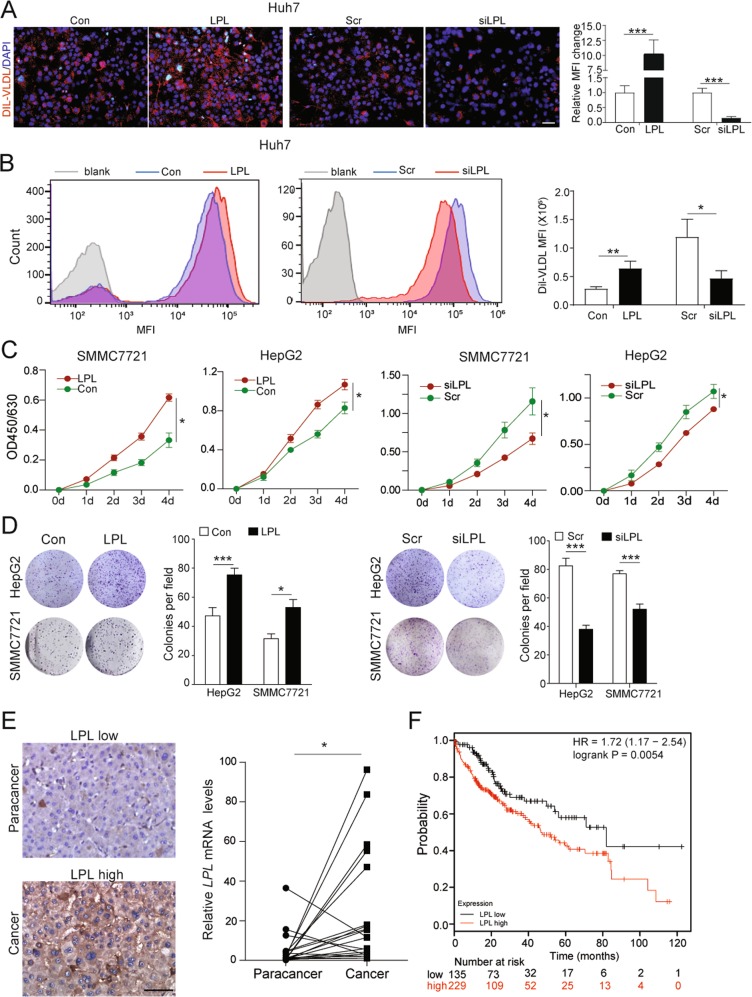
LPL inhibits exogenous lipid uptake and cell proliferation of HCC cells. **a** Dil-VLDL accumulated in Huh7 cells with overexpression or knockdown of ZHX2. *n* = 4; ***p < 0.001. **b** Flow cytometry was used to analyze Dil-VLDL uptake in Huh7 cells with ZHX2 overexpression or knockdown. *n* = 3; *p *<* 0.05, **p *<* 0.01. **c**, **d** SMMC7721 and HepG2 cells with LPL overexpression or knockdown were used to measure cell proliferation (**c**) and colony formation (**d**). *n* ≥ 4; *p *<* 0.05, ***p *<* 0.001. **e** LPL expression was investigated in an HCC cohort. Representative IHC images of LPL staining in the tissue microarray were shown on the left panel. Statistic data of LPL mRNA levels in 20 pairs of samples were shown on the right panel. *p *<* 0.05. **f** High levels of LPL were associated with poor overall survival of 364 HCC patients from a KM plot database

To evaluate the role of LPL in NAFLD progression, Dil-VLDL was used to treated HCC cells with LPL manipulation. As shown in Fig. 5a, ectopic expression of LPL increased uptake of Dil-VLDL, while knockdown of endogenous LPL decreased uptake of Dil-VLDL in Huh7 cells, indicating the involvement of LPL in uptake of lipoproteins. This result was further confirmed by flow cytometry (Fig. 5b). Furthermore, in vivo study showed that liver-specific overexpression of LPL increased levels of TG and cholesterol in the mice livers and promoted NAFLD progression (Fig. S3A). Our findings are in line with a recent study reporting that LPL simultaneously bound to lipoproteins and cell surface proteins, leading to uptake of lipoproteins [25].

To investigate the role of LPL in HCC development, cell proliferation was measured in HCC cell lines with modulated LPL expression. As shown in Fig. 5c, d, overexpression of LPL dramatically increased HCC cell proliferation and colony formation, while knockdown of LPL clearly inhibited HCC cell proliferation and colony formation. Furthermore, LPL expression, both in mRNA and protein level, was significantly higher in HCC tissues compared with paracancers (Fig. 5e and Table S1). And the levels of LPL expression in stages III/IV of HCC were higher than stages I/II of HCC (Table S2). Consistently, KM plotter analysis (www.kmplot.com) showed a negative correlation of LPL expression with overall survival in HCC patients (Fig. 5f) [26]. Taken together, all these data suggest that, as the transcriptional target of ZHX2, LPL not only mediates lipids uptake of HCC cell lines but also promotes HCC tumor growth.

### ZHX2 blocks lipids uptake and NAFLD progression by repressing LPL

**Fig. 6:**
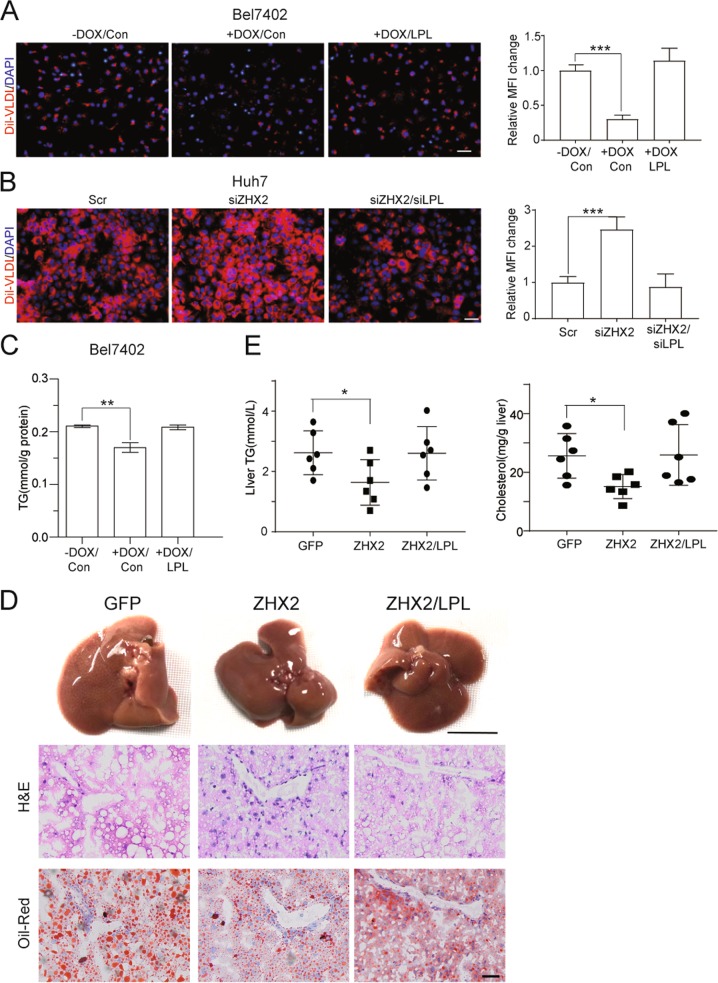
ZHX2 inhibits the progression of NAFLD by repressing LPL expression. **a**, **b** Dil-VLDL accumulated in Huh7 and Bel7402 cells after transfected with indicated plasmids. *n* = 4; ***p *<* 0.001. **c** Total TG levels were measured in Bel7402 cells with ZHX2 and LPL overexpression, simultaneously or individually. *n* = 4; **p *<* 0.01. **d** AAV-ZHX2 and AAV-LPL were injected in mice *via* tail vein simultaneously or individually. Then the mice were fed with HFD to induce fatty liver. The representative images of mice livers, H&E, and Oil Red O staining were shown. **e** Mice liver tissues were collected and homogenized to measure total levels of TG and cholesterol. *n* = 6; *p *<* 0.05

To define the role of LPL in ZHX2-inhibited lipid deposition, the lipid content was measured in HCC cells treated with exogenous lipid. As expected, ZHX2 decreased uptake of Dil-VLDL, while ectopic expression of LPL abolished the inhibition of ZHX2 on the uptake of Dil-VLDL in Bel7402 (Fig. 6a). Reciprocally, knockdown of endogenous LPL clearly reversed ZHX2 knockdown-enhanced Dil-VLDL uptake in Huh7 cells (Fig. 6b). Furthermore, LPL overexpression ameliorated ZHX2-decreased TG accumulation in Bel7402 cells (Fig. 6c). Taken together, all the data suggest that ZHX2 inhibits accumulation of lipids in HCC cells by repressing LPL expression.

The role of LPL in ZHX2-mediated retardation of NAFLD was further investigated using murine models. Thus, mice were injected with AAV-ZHX2 and/or AAV-LPL via tail vein, and then fed with HFD. As shown in Fig. 6d, H&E and Oil Red O staining demonstrated that ZHX2 overexpression significantly inhibited hepatic lipid deposition and maintained normal hepatic structure, which were significantly dampened by overexpression of LPL. Also, ZHX2 overexpression decreased levels of hepatic TG and cholesterol, while ectopic LPL expression reversed ZHX2-decreased TG and cholesterol in liver homogenizes (Fig. 6e). Similar results were also achieved in MCD-diet fed mice with ZHX2 and/or LPL expression (Fig. S3B). All these results demonstrate that ZHX2 inhibits lipid deposition in liver and hampers the progression of NAFLD by suppressing LPL expression.

### ZHX2 inhibits cell proliferation and liver tumor growth by repressing LPL

**Fig. 7:**
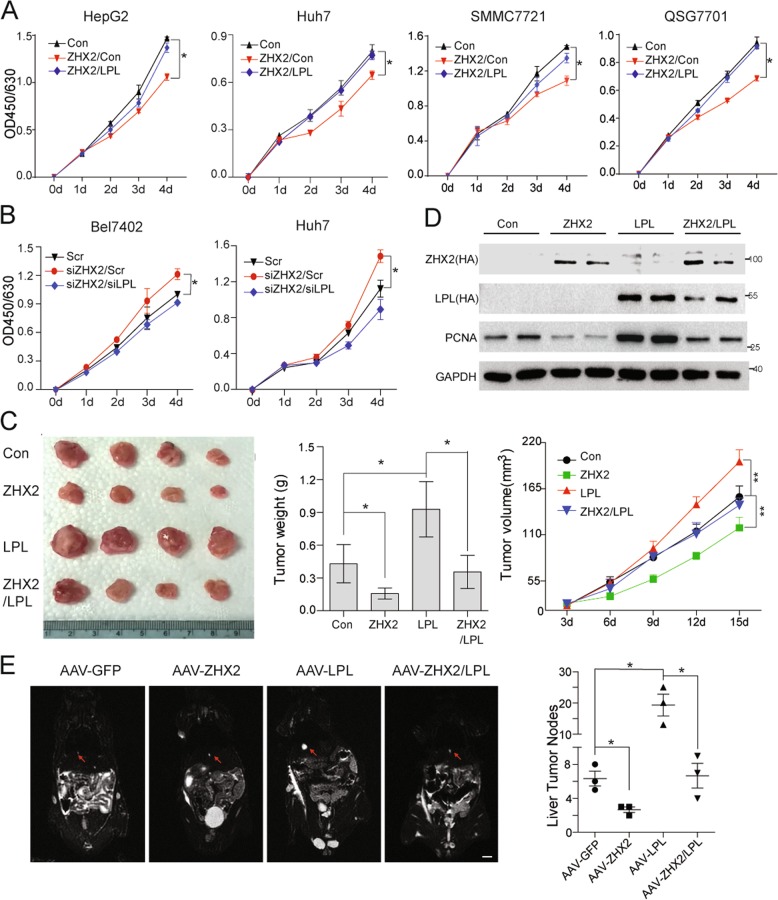
ZHX2 inhibits cell proliferation and tumor growth by repressing LPL. Cell proliferation was measured in HepG2, Huh7, SMMC7721 and QSG7701 cells with overexpression of ZHX2 and LPL (**a**) and Bel7402 and Huh7 cells with knockdown of ZHX2 and LPL (**b**), using a CCK8 assay kit. *n* ≥ 3; *p *<* 0.05. **c**, **d** H22 cells were subcutaneously injected into nude mice to form xenograft tumor. Afterwards, ZHX2 and LPL expression vectors were injected every two days in the xenograft tumors, simultaneously or individually. Images, weight and growth curve of xenograft tumors were presented to illustrate the impact of ZHX2 and LPL expression on carcinogenesis (**c**). *n* ≥ 5; *p *<* 0.05, **p *<* 0.01. ZHX2 and LPL expression and cell proliferation marker PCNA were determined by Western blot. Representative blotting were shown from xenograft experiments (**d**). **e** C57BL/6 mice were injected with AAV-GFP, AAV-ZHX2, AAV-LPL, and AAV-ZHX2 plus AAV-LPL *via* the tail vein, then the mice were used to induce liver tumors by STZ–HFD. Tumor nodes on the mice lives were identified by the magnetic resource imaging using 3.0 T NMR (left panel). At the end of experiment, mice were euthanized to calculate numbers of tumor nodes in the livers (right panel). Mean ± SEM, *n* = 3; *p *<* 0.05

Since ZHX2 was negatively associated with LPL, we hypothesized that ZHX2 might exert its HCC suppressor function through repression of LPL. Hence, cell proliferation was measured in HCC cells with manipulated expression of ZHX2 and LPL. Figure 7a showed that ZHX2 inhibited cell proliferation, and LPL overexpression reversed ZHX2’s inhibitory effect. Reciprocally, ZHX2 knockdown significantly promoted cell growth, which was markedly reversed by LPL knockdown in Huh7 and Bel7402 cells (Fig. 7b). Furthermore, ZHX2 overexpression suppressed the growth of xenograft tumors, while ectopic expression of LPL enhanced tumor growth, and clearly reversed the ZHX2-inhibited xenograft tumor growth (Fig. 7c). Western blotting of PCNA confirmed the impacts of LPL on ZHX2-mediated inhibition of cell proliferation (Fig. 7d).

Aiming to further establish the concept of ZHX2/LPL axis in NAFLD–HCC progression, ZHX2 (AAV-ZHX2) and LPL (AAV-LPL) were introduced into STZ–HFD mice either separately or simultaneously. As expected, the number of tumor nodes in mice injected with AAV-ZHX2 were significantly less compared with the control mice, suggesting that ZHX2 suppresses the progression of NAFLD to HCC. Notably, LPL overexpression induced by AAV-LPL not only increased the number of tumor nodes but also reversed the inhibitory effect of ZHX2 on tumor formation in STZ–HFD mice (Fig. 7e). These results were also supported by IHC staining of Ki67. ZHX2 overexpression decreased Ki67^+^ cells, while ectopic LPL elevated Ki67^+^ tumor cells and restored Ki67 expression in ZHX2 tumor cells (Fig. S3C). Collectively, all above data suggest that ZHX2 hampers NAFLD–HCC progression by suppressing LPL expression.

### ZHX2 negatively associates with LPL expression in human HCC samples

**Fig. 8:**
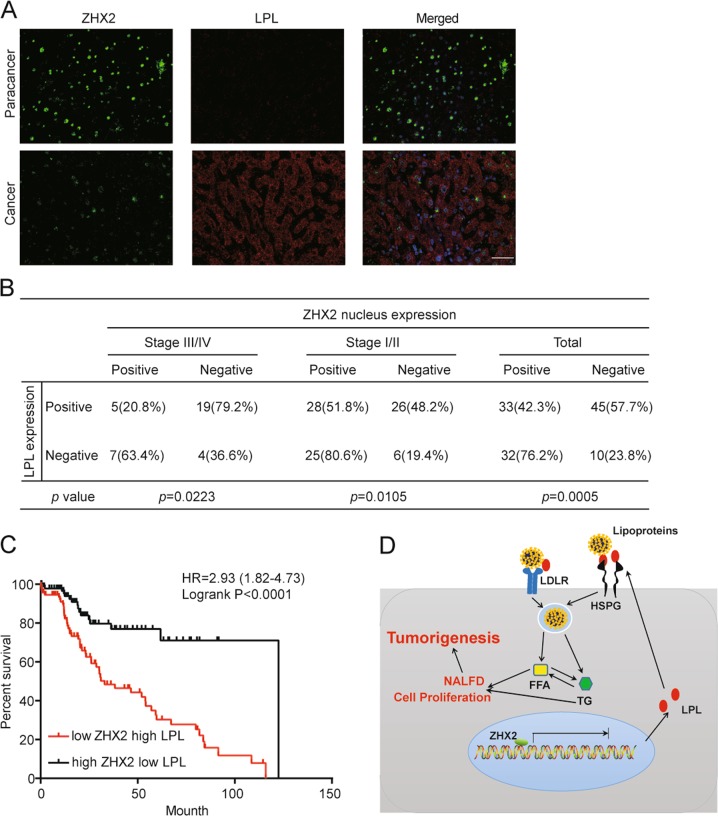
ZHX2-LPL axis represents a negative association that controls HCC progression. **a** Co-IF staining of LPL and ZHX2 in paired HCC tissues. Representative images of paracancer tissues (upper panel) and cancer tissues (lower panel) were shown. **b** Statistic analysis of ZHX2 and LPL correlation in different stages of HCC tissues. **c** HCC patients were grouped by high ZHX2 & low LPL and low ZHX2 & high LPL, and the overall survivals of the two groups were analyzed using Logrank analysis. **d** Schematic model depicts that ZHX2 inhibits LPL expression at transcriptional levels, which in turn reduces LPL-mediated lipid uptake, inhibits NAFLD and cell proliferation, and consequently hampers HCC progression

In order to further validate the involvement of ZHX2/LPL axis in human HCC tissues, IHC staining of ZHX2, and LPL were performed using serial sections from HCC tissues. Consistent with our previous report [15], expression levels of ZHX2 in stages III/IV of HCC were lower than stages I/II of HCC (Table S3). Interestingly, co-IF staining showed that paracancer tissues with higher ZHX2 nuclear expression displayed lower LPL levels. Conversely, cancer tissues with lower ZHX2 expression had higher LPL levels (Fig. 8a). Among the 120 HCC specimens, positive ZHX2 and LPL staining was found in 54.2% (65 of 120) and 65% (78 of 120) of tumors, respectively. Statistical analysis revealed the significant negative correlation between ZHX2 and LPL expression in total specimens, stages I/II and III/IV of HCC (Fig. 8b). In addition, ZHX2 truncated protein ZHX2(242–446), which contains the HD1 and HD2 with nuclear localization signal (NLS) [15], inhibited LPL expression at protein levels. While ZHX2(242–439), which contains HD1 and HD2 without NLS [15], had no effect on LPL expression (Fig. S3D). Furthermore, survival analysis of HCC patients suggested that patients with high ZHX2 but low LPL had a significantly better prognosis in survival, and that patients with low ZHX2 but high LPL had much poorer overall survival (Fig. 8c). Therefore, our results indicated that ZHX2 is negatively correlated with LPL expression in HCC, and that high ZHX2 expression is beneficial for the survival of HCC patients.

## Discussion

Liver plays a central role in lipid homeostasis. Both NAFLD and HCC are lipid metabolism disorder-associated liver diseases [1, 5], to which increased accumulation of lipids in hepatocytes is a leading cause [27]. It is reported that altered expression of several genes encoding FATPs, CD36, SREBP1, and PPARγ may influence lipid accumulation in hepatocytes [28]. However, key regulators responsible for abnormal hepatic lipid accumulation during NAFLD–HCC are largely unknown. Here, we showed that ZHX2 expression was reduced in fatty liver tissues and altered ZHX2 disturbed lipid homeostasis of hepatocytes and NAFLD–HCC progression. To our best knowledge, this is the first study revealing the critical roles of ZHX2 in hepatic lipid deposition and NAFLD–HCC progression. Our findings highlighted that ZHX2 is critical regulator of lipid homeostasis for both normal and malignant hepatocytes.

Many studies including ours demonstrate ZHX2 as an HCC-associated tumor suppressor [9, 12, 15, 29]. ZHX2 inhibits HCC growth through suppression of HCC biomarkers (AFP, GPC3, and H19) and cell cycle genes (Cyclin A and Cyclin E) [12, 13, 15]. Recent research reported that ZHX2 regulates plasma lipids homeostasis [17], indicating the potential involvement of ZHX2 in lipid metabolism and related diseases, such as NAFLD and HCC. However, till now, the exact role of ZHX2 in NAFLD and HCC are unknown. In the present study, we have proved that suppressed hepatic lipid accumulation and protected mice from MCD and HFD induced NAFLD (Figs. 1 and 2). Lipids play important roles in tumor biology, including energy supply, cell signaling transduction, and cell membrane formation [30]. Our data demonstrated that ZHX2 controlled exogenous lipids uptake in HCC cells, thus limited energy resources of HCC cells to inhibit its proliferation (Fig. 3). Furthermore, in vivo data showed that ZHX2 markedly suppressed NAFLD–HCC progression in STZ–HFD mice model (Fig. 7). Accumulating evidence showed that high proliferative tumor cells increase uptake of exogenous lipids [31]. Targeting the fatty acid transporter CD36 almost completely inhibited tumor metastasis in mice model [22]. Our finding suggests ZHX2 is an useful target for blocking exogenous lipid uptake in HCC. It has been well known that *de novo* lipid synthesis is critical for tumor cells to meet their high proliferative demands [30]. It is unclear whether ZHX2 inhibits *de novo* lipid synthesis to exert its tumor suppressor function in HCC, which will be an interest research direction.

LPL is a key enzyme of lipid metabolism, and is synthesized mainly in adipose tissue, skeletal muscles, and mammary gland [22]. LPL regulates lipid metabolism mainly through two ways. On the one hand, LPL catalyzes hydrolysis of TG, the main component of chylomicrons and VLDL [25]. On the other hand, non-catalytic function of LPL involved in the uptake of lipoprotein remnant via its functional coordination with HSPG, LDL receptors and VLDL receptors [25, 32]. In the hepatocytes, LPL is synthesized at fetal liver, then turned dim soon after birth [25], similar to the expression pattern of AFP, which is the first identified target of ZHX2 and promotes HCC development [12, 33]. Here, we have identified LPL as the novel target of ZHX2 (Fig. 4). This is consistent with earlier observations that LPL expression was increased in BALB/cJ mice with a mutated *Zhx2* gene and decreased in that with transgene *Zhx2* [17].

LPL plays important roles in lipid metabolisms and related diseases. LPL deficiency led to hyperlipidemia [34, 35]. Recent study reported that LPL promoted breast cancer cell growth and survival [23]. However, its function in HCC is still not clear. In this study, we showed that LPL enhanced exogenous lipid uptake, prompted proliferation of HCC cells and negatively associated with survival of patients (Fig. 5). All these data pinpoint the critical role of LPL in HCC progression. It should be also stressed that ZHX2 inhibited exogenous lipid uptake and NAFLD–HCC progression via repressing LPL (Figs. 6 and 7). This is supported by the clinical data, showing low ZHX2 but high LPL expression had a poorer overall survival as compared with those with high ZHX2 but low LPL levels (Fig. 8). Therefore, ZHX2-LPL axis has emerged as a novel regulatory mechanism of NAFLD–HCC progression. Of note, we could not exclude LPL’s catalytic function in HCC development. Whether LPL catalytic function also play a role in HCC development is not clear. This will be of interest to investigate in future studies.

In conclusion, ZHX2 transcriptionally represses LPL expression, and subsequently inhibits exogenous lipid uptake of hepatocytes. The present study has established the ZHX2-LPL axis that maintains hepatocytic lipid homeostasis, hampers NAFLD development and NAFLD–HCC progression, and suppresses HCC tumor growth. Our findings not only provide new insights in the mechanisms of NAFLD–HCC progression, but also foster a novel therapeutic strategy for NAFLD-associated HCC management.

## Supplementary information

Supplement Table 1

Supplement Table 2

Supplement Table 3

Supplement Figure 1

Supplement Figure 2

Supplement Figure 3

Supplement Figure legend

Supplement Method

## References

[1] Baffy G, Brunt EM, Caldwell SH (2012). Hepatocellular carcinoma in non-alcoholic fatty liver disease: an emerging menace. J Hepatol.

[2] Sung H, Siegel RL, Torre LA, Pearson-Stuttard J, Islami F, Fedewa SA, et al. Global patterns in excess body weight and the associated cancer burden. CA Cancer J Clin. 2019;69:88–112. 10.3322/caac.21499 30548482

[3] Lambert JE, Ramos-Roman MA, Browning JD, Parks EJ (2014). Increased de novo lipogenesis is a distinct characteristic of individuals with nonalcoholic fatty liver disease. Gastroenterology.

[4] Stickel F, Hellerbrand C (2010). Non-alcoholic fatty liver disease as a risk factor for hepatocellular carcinoma: mechanisms and implications. Gut.

[5] Jiang CM, Pu CW, Hou YH, Chen Z, Alanazy M, Hebbard L (2014). Non alcoholic steatohepatitis a precursor for hepatocellular carcinoma development. World J Gastroenterol.

[6] Link T, Iwakuma T (2017). Roles of p53 in extrinsic factor-induced liver carcinogenesis. Hepatoma Res.

[7] Menendez JA, Lupu R (2007). Fatty acid synthase and the lipogenic phenotype in cancer pathogenesis. Nat Rev Cancer.

[8] Horie Y, Suzuki A, Kataoka E, Sasaki T, Hamada K, Sasaki J (2004). Hepatocyte-specific Pten deficiency results in steatohepatitis and hepatocellular carcinomas. J Clin Invest.

[9] Kawata H, Yamada K, Shou Z, Mizutani T, Yazawa T, Yoshino M (2003). Zinc-fingers and homeoboxes (ZHX) 2, a novel member of the ZHX family, functions as a transcriptional repressor. Biochemical J.

[10] Perincheri S, Dingle RW, Peterson ML, Spear BT (2005). Hereditary persistence of alpha-fetoprotein and H19 expression in liver of BALB/cJ mice is due to a retrovirus insertion in the Zhx2 gene. Proc Natl Acad Sci USA.

[11] Morford LA, Davis C, Jin L, Dobierzewska A, Peterson ML, Spear BT (2007). The oncofetal gene glypican 3 is regulated in the postnatal liver by zinc fingers and homeoboxes 2 and in the regenerating liver by alpha-fetoprotein regulator 2. Hepatology.

[12] Shen H, Luan F, Liu H, Gao L, Liang X, Zhang L (2008). ZHX2 is a repressor of alpha-fetoprotein expression in human hepatoma cell lines. J Cell Mol Med.

[13] Luan F, Liu P, Ma H, Yue X, Liu J, Gao L (2014). Reduced nucleic ZHX2 involves in oncogenic activation of glypican 3 in human hepatocellular carcinoma. Int J Biochem Cell Biol.

[14] Lv Z, Zhang M, Bi J, Xu F, Hu S, Wen J (2006). Promoter hypermethylation of a novel gene, ZHX2, in hepatocellular carcinoma. Am J Clin Pathol.

[15] Yue X, Zhang Z, Liang X, Gao L, Zhang X, Zhao D (2012). Zinc fingers and homeoboxes 2 inhibits hepatocellular carcinoma cell proliferation and represses expression of Cyclins A and E. Gastroenterology.

[16] Ma H, Yue X, Gao L, Liang X, Yan W, Zhang Z (2015). ZHX2 enhances the cytotoxicity of chemotherapeutic drugs in liver tumor cells by repressing MDR1 via interfering with NF-YA. Oncotarget.

[17] Gargalovic PS, Erbilgin A, Kohannim O, Pagnon J, Wang X, Castellani L (2010). Quantitative trait locus mapping and identification of Zhx2 as a novel regulator of plasma lipid metabolism. Circ Cardiovasc Genet.

[18] Du X, Wu Z, Xu Y, Liu Y, Liu W, Wang T, et al. Increased Tim-3 expression alleviates liver injury by regulating macrophage activation in MCD-induced NASH mice. Cell Mol Immunol. 2019;16:878–86. 10.1038/s41423-018-0032-0 PMC6828758 29735977

[19] Creasy KT, Jiang J, Ren H, Peterson ML, Spear BT (2016). Zinc fingers and homeoboxes 2 (Zhx2) regulates sexually dimorphic cyp gene expression in the adult mouse liver. Gene Expr.

[20] Fujii M, Shibazaki Y, Wakamatsu K, Honda Y, Kawauchi Y, Suzuki K (2013). A murine model for non-alcoholic steatohepatitis showing evidence of association between diabetes and hepatocellular carcinoma. Med Mol Morphol.

[21] Diehl AM, Goodman Z, Ishak KG (1988). Alcohollike liver disease in nonalcoholics. A clinical and histologic comparison with alcohol-induced liver injury. Gastroenterology.

[22] Pascual G, Avgustinova A, Mejetta S, Martin M, Castellanos A, Attolini CS (2017). Targeting metastasis-initiating cells through the fatty acid receptor CD36. Nature.

[23] Kuemmerle NB, Rysman E, Lombardo PS, Flanagan AJ, Lipe BC, Wells WA (2011). Lipoprotein lipase links dietary fat to solid tumor cell proliferation. Mol Cancer Ther.

[24] Wu X, Sakata N, Dixon J, Ginsberg HN (1994). Exogenous VLDL stimulates apolipoprotein B secretion from HepG2 cells by both pre- and post-translational mechanisms. J Lipid Res.

[25] Mead JR, Irvine SA, Ramji DP (2002). Lipoprotein lipase: structure, function, regulation, and role in disease. J Mol Med (Berl).

[26] Szasz AM, Lanczky A, Nagy A, Forster S, Hark K, Green JE (2016). Cross-validation of survival associated biomarkers in gastric cancer using transcriptomic data of 1065 patients. Oncotarget.

[27] Sanyal AJ, Brunt EM, Kleiner DE, Kowdley KV, Chalasani N, Lavine JE (2011). Endpoints and clinical trial design for nonalcoholic steatohepatitis. Hepatology.

[28] Greco D, Kotronen A, Westerbacka J, Puig O, Arkkila P, Kiviluoto T (2008). Gene expression in human NAFLD. Am J Physiol Gastrointest Liver Physiol.

[29] Liu Y, Ma D, Ji C (2015). Zinc fingers and homeoboxes family in human diseases. Cancer Gene Ther.

[30] Schulze A, Harris AL (2012). How cancer metabolism is tuned for proliferation and vulnerable to disruption. Nature.

[31] Beloribi-Djefaflia S, Vasseur S, Guillaumond F (2016). Lipid metabolic reprogramming in cancer cells. Oncogenesis.

[32] Hu L, van der Hoogt CC, Espirito Santo SM, Out R, Kypreos KE, van Vlijmen BJ (2008). The hepatic uptake of VLDL in lrp-ldlr-/-vldlr-/- mice is regulated by LPL activity and involves proteoglycans and SR-BI. J Lipid Res.

[33] Pachnis V, Belayew A, Tilghman SM (1984). Locus unlinked to alpha-fetoprotein under the control of the murine raf and Rif genes. Proc Natl Acad Sci USA.

[34] Yang WS, Nevin DN, Peng R, Brunzell JD, Deeb SS (1995). A mutation in the promoter of the lipoprotein lipase (LPL) gene in a patient with familial combined hyperlipidemia and low LPL activity. Proc Natl Acad Sci USA.

[35] Fager G, Semb H, Enerback S, Olivecrona T, Jonasson L, Bengtsson-Olivecrona G (1990). Hyperlipoproteinemia type I in a patient with active lipoprotein lipase in adipose tissue and indications of defective transport of the enzyme. J Lipid Res.

