# Supplementary material for: Tumor suppressor ZHX2 inhibits NAFLD–HCC progression via blocking LPL-mediated lipid uptake
Source: Cell Death Differ. 2019 Nov 18;27(5):1693–708. doi: 10.1038/s41418-019-0453-z (PMC7206072; doi:10.1038/s41418-019-0453-z)
Supplement: Supplementary file 1 — Supplement Table 1 [file 41418_2019_453_MOESM1_ESM.doc]

**Table S1. Immunohistochemical staining of LPL expression in HCC clinical specimens.**

|  |  | Number  of  case | LPL expression  (Cytoplasmic staining) | | |
| --- | --- | --- | --- | --- | --- |
|  |  |  | Positive  (4-12) | Negative  (0-3) | Mean ± SD  (range) |
| All specimens | Noncancer | 75 | 45  (60%) | 30  (40%) | 3.68 ± 2.04  (0-10) |
|  | Cancer | 75 | 67  (89.3%) | 8  (10.7%) | 6.74 ± 2.75  (2-12) |
|  | *p* value |  | *p* < 0.0001^a^ | | 0.01 < *p* < 0.05^b^ |

^a^ *p* values were obtained from the *Chi-square test*.

^b^ *p* values were obtained from the *non-parametric test*.
