# Supplementary material for: Tumor suppressor ZHX2 inhibits NAFLD–HCC progression via blocking LPL-mediated lipid uptake
Source: Cell Death Differ. 2019 Nov 18;27(5):1693–708. doi: 10.1038/s41418-019-0453-z (PMC7206072; doi:10.1038/s41418-019-0453-z)
Supplement: Supplementary file 2 — Supplement Table 2 [file 41418_2019_453_MOESM2_ESM.doc]

**Table S2. LPL expression in different stages of HCC clinical specimens.**

| Stage of HCC | Number of case | LPL expression | | |
| --- | --- | --- | --- | --- |
|  |  | Positive  (4-12) | Negative  (0-3) | Mean ± SD  (range) |
| Ⅰ/Ⅱ | 85 | 54  (63.53%) | 31  (36.47%) | 5.72 ± 0.36  (0-12) |
| Ⅲ/Ⅳ | 35 | 24  (68.57%) | 11  (31.43%) | 7.14 ± 0.51  (0-12) |
| *p* value | | *P*=0.6766^a^ | | *p* =0.029^b^ |

^a^ *p* values were obtained from the *fisher’s exact test*.

^b^ *p* values were obtained from the *non-parametric test*.
