# Supplementary material for: Tumor suppressor ZHX2 inhibits NAFLD–HCC progression via blocking LPL-mediated lipid uptake
Source: Cell Death Differ. 2019 Nov 18;27(5):1693–708. doi: 10.1038/s41418-019-0453-z (PMC7206072; doi:10.1038/s41418-019-0453-z)
Supplement: Supplementary file 3 — Supplement Table 3 [file 41418_2019_453_MOESM3_ESM.doc]

**Table S3. ZHX2 expression in different stages of HCC clinical specimens.**

| Stage of HCC | Number of case | ZHX2 expression | | |
| --- | --- | --- | --- | --- |
|  |  | Positive  (4-12) | Negative  (0-3) | Mean ± SD  (range) |
| Ⅰ/Ⅱ | 85 | 45  (60.81%) | 29  (39.19%) | 4.78 ± 0.30  (0-12) |
| Ⅲ/Ⅳ | 35 | 12  (34.29%) | 23  (65.71%) | 3.26 ± 0.46  (0-12) |
| *p* value | | *P*=0.0135^a^ | | *p* =0.0064^b^ |

^a^ *p* values were obtained from the *fisher’s exact test*.

^b^ *p* values were obtained from the *non-parametric test*.
