# Supplementary figures and images for: Tumor suppressor ZHX2 inhibits NAFLD–HCC progression via blocking LPL-mediated lipid uptake
Source: Cell Death Differ. 2019 Nov 18;27(5):1693–708. doi: 10.1038/s41418-019-0453-z (PMC7206072; doi:10.1038/s41418-019-0453-z)

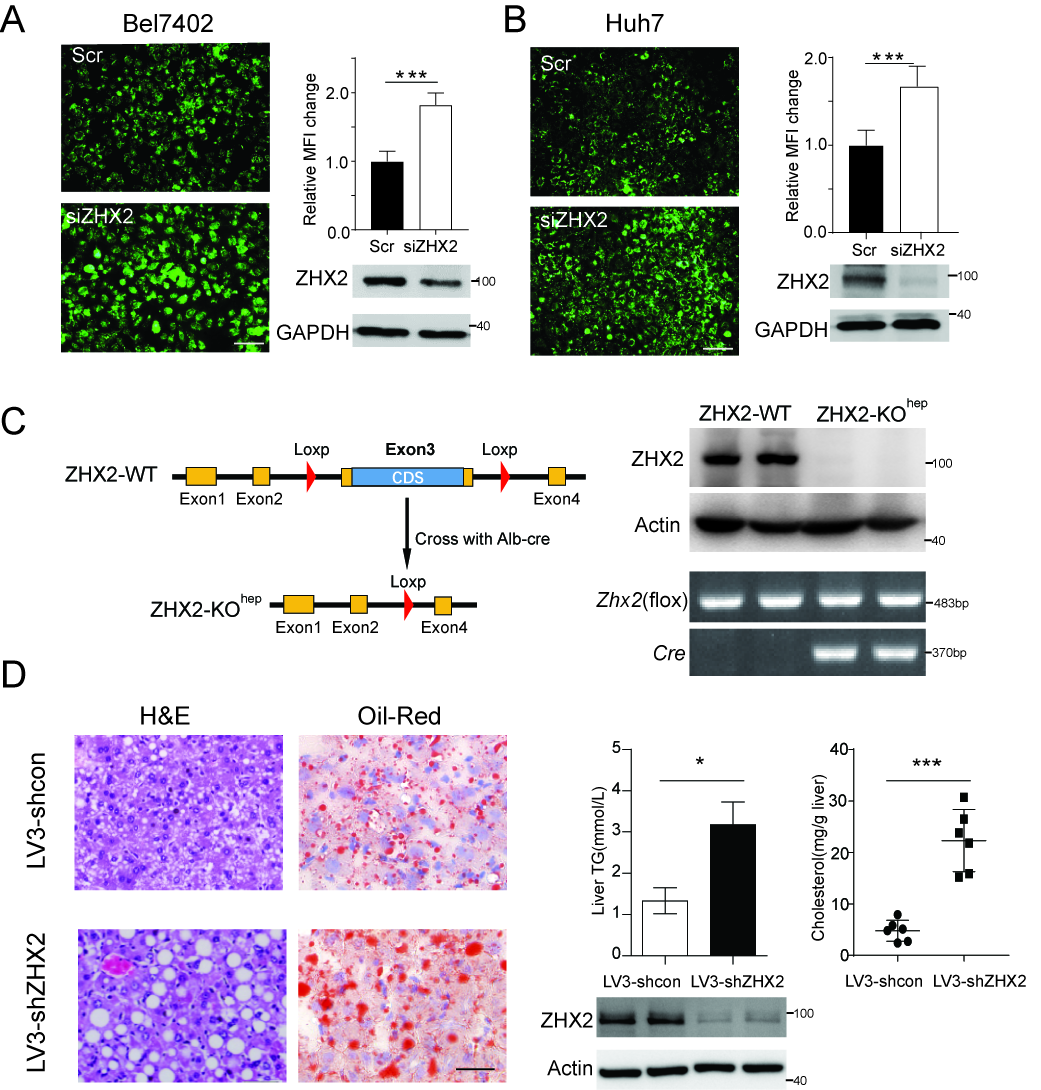

Supplement: Supplementary file 4 — Supplement Figure 1 [file 41418_2019_453_MOESM4_ESM.tif]

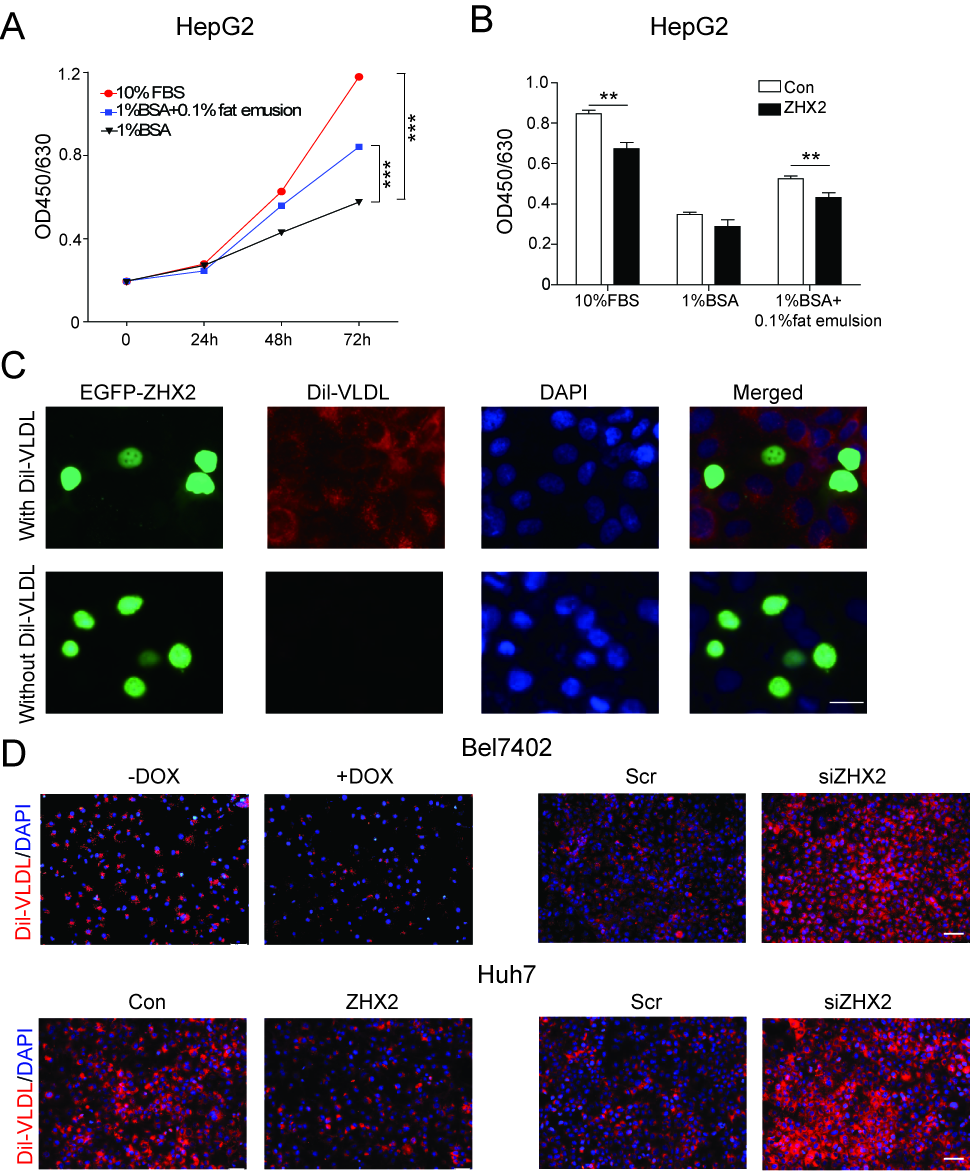

Supplement: Supplementary file 5 — Supplement Figure 2 [file 41418_2019_453_MOESM5_ESM.tif]

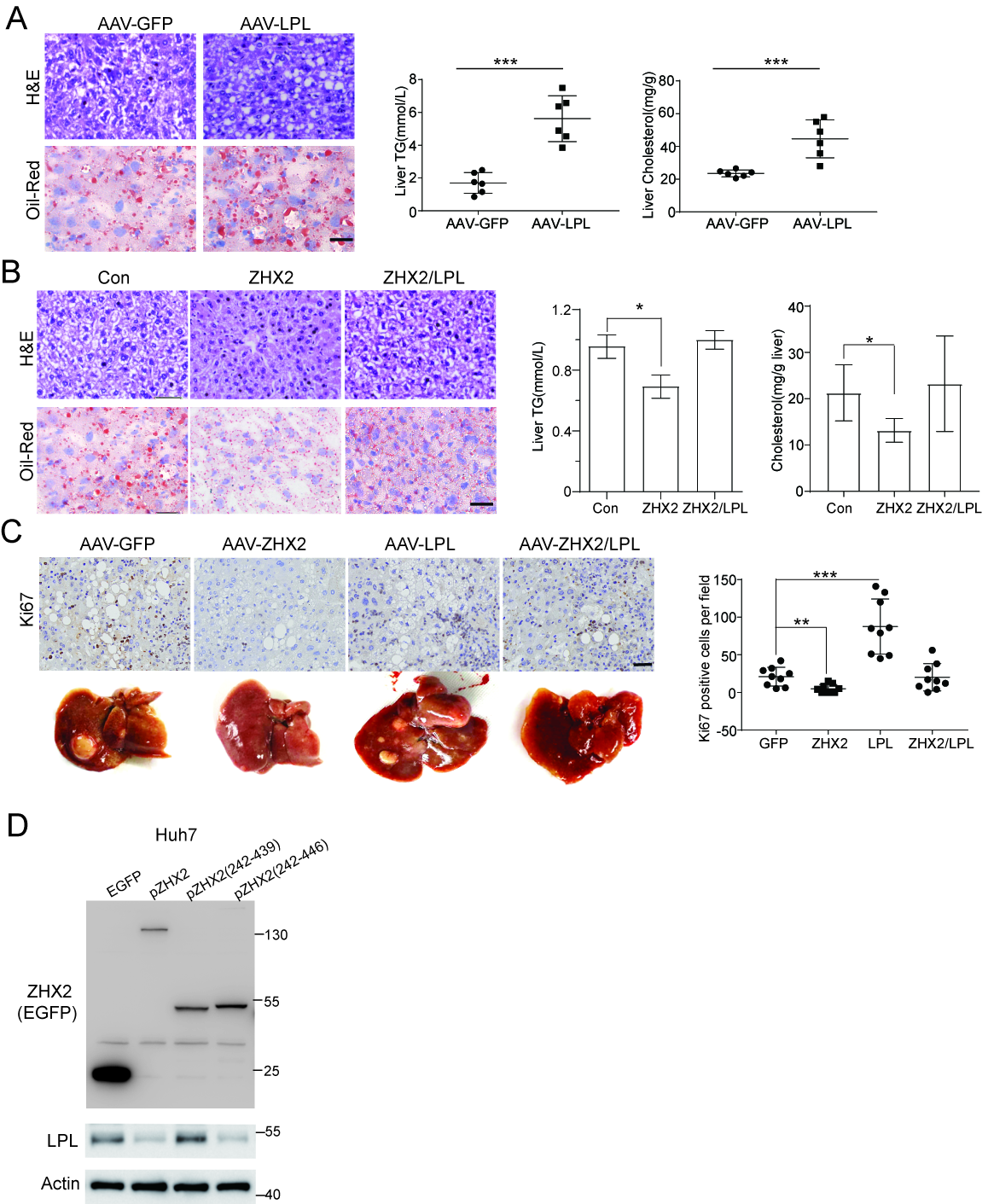

Supplement: Supplementary file 6 — Supplement Figure 3 [file 41418_2019_453_MOESM6_ESM.tif]
