# Supplementary material for: Tumor suppressor ZHX2 inhibits NAFLD–HCC progression via blocking LPL-mediated lipid uptake
Source: Cell Death Differ. 2019 Nov 18;27(5):1693–708. doi: 10.1038/s41418-019-0453-z (PMC7206072; doi:10.1038/s41418-019-0453-z)
Supplement: Supplementary file 7 — Supplement Figure legend [file 41418_2019_453_MOESM7_ESM.docx]

**Supplement figure legend**

**Supplement Figure 1.**

**(*A*** and ***B*)** ZHX2 siRNA-transfected Bel7402 and Huh7 cells were used to induce lipid deposition by using fat emulsion treatment. The knockdown efficiency was confirmed by western blot. Bodipy staining was used to illustrate lipid deposition in the cells. The representative images were showed on the left panels. Statistics of Bodipy intensity was shown on the right panels. **(*C*)** The diagram shows the strategy of ZHX2 specific knockdown in hepatocytes. Liver tissues were collected to confirm the murine model by Western blot. PCR was performed using the template DNA extracted from mice tail. Mice homozygous for floxed *Zhx2* allele with or without *Alb-Cre* transgene (designated as ZHX2-KO^hep^ or ZHX2-WT). **(*D*)** The mice with specific knockdown of ZHX2 via lentivirus armed with ZHX2 shRNA in the livers were used to induce NAFLD by feeding with MCD diet. ZHX2 specifically knockdown in mouse liver was confirmed by Western blot. The representative images of H&E and Oil Red O staining were shown on the left panel. Right panel showed the statistic results of TG and cholesterol levels in liver homogenate. *n≥4*; * *p<0.05*.

**Supplement Figure 2.**

**(*A*)**  HepG2 cells were cultured in the low glucose medium with 10% FBS or with 1% fatty acids-free BSA plus 0.1% fat emulsion. Cell proliferation was assessed using a CCK8 assay kit. Mean±SED; *n≥3*; *** *p*<0.001. **(*B*)** HepG2 cells with or without ZHX2 overexpression were cultured in low glucose medium with 10%FBS, 1% fatty acids-free BAS or 1% fatty-free BSA plus 0.1% fat emulsion to measure cell proliferation. Mean±SED; *n≥3*; ** *p*<0.01. **(*C*)** Huh7 cells transfected with EGFP-tagged ZHX2 and cultured in the medium with or without Dil-VLDL. ZHX2 localization and VLDL intensity were shown by the representative images. **(*D*)** Bel7402 and Huh7 cells with ZHX2 overexpression or knockdown were treated with Dil-VLDL. The representative images were used to show accumulation of Dil-VLDL in the cells.

**Supplement Figure 3.**

**(*A*)** The mice were specifically overexpressed LPL in the livers through injection of AAV-LPL *via* tail vein, then fed with HFD to induce NAFLD. The representative images of Oil Red O and H&E staining were shown on left panel. The analysis of TG and cholesterol levels in the mice livers were shown on right panel. Mean±SED; *n=6;** *p<0.05*. **(*B*)** Control, ZHX2 and LPL vectors were injected, alone or in combination, into the mice *via* the tail vein. Afterwards, the mice were fed with MCD diet to induce fatty liver. The representative images of Oil Red O and H&E staining were shown on left panel. Mouse liver tissues were collected and homogenized to measure the total levels of TG and cholesterol. Mean±SED; *n=6;** *p<0.05*. **(*C*)** ZHX2 and LPL expression were manipulated in mice livers. Then the mice were induced liver tumors by STZ-HFD method. Ki67 staining of liver sections and whole mice livers were shown on left panel. The statistic results of Ki67 positives cells were analyzed on right panel. Mean±SED; *n=9*; **, *p<0.01*. ***, *p<0.001*. **(*D*)** Full length ZHX2 vector (pZHX2), ZHX2 truncated vectors [pZHX2(242-446) and pZHX2(242-439)] and control vector (EGFP) were transfected in Huh7 cells. Then total protein of transfected Huh7 cells were collected to detect LPL by Western blot.
