# Supplementary material for: Tumor suppressor ZHX2 inhibits NAFLD–HCC progression via blocking LPL-mediated lipid uptake
Source: Cell Death Differ. 2019 Nov 18;27(5):1693–708. doi: 10.1038/s41418-019-0453-z (PMC7206072; doi:10.1038/s41418-019-0453-z)
Supplement: Supplementary file 8 — Supplement Method [file 41418_2019_453_MOESM8_ESM.docx]

**Supplement Methods and Materials**

***Cells and reagents***

The human HCC cell lines HepG2, Huh7, SMMC7721, QSG7701 and Bel7402 were purchased from the Cell Bank of the Chinese Academy of Science (Shanghai, China), and cultured in DMEM (Thermo Fisher Scientific) with 10% FBS. The Doxycycline (Dox)-induced Bel7402-ZHX2-Teton cell line was established by using Tet-On Inducible Expression System (Clontech Laboratories).

The commercial antibodies used in this study include anti-ZHX2 (1:5000; Proteintech), anti-LPL (1:1000; Abcam), anti-β-actin (1:5000; Proteintech), anti-GAPDH (1:5000; Proteintech), anti-Flag (1:1000; Sigma), and anti-HA (1:2000; Sigma).

Dil-VLDL was purchased from Kalen Biomedical. BODIPY FL dye was purchased from Thermo Fisher Scientific. Charcoal Adsorbed Serum was purchased from Biological Industries (BI). Oil Red O (ORO), oleic acid (OA), Dox, fatty acid-free BSA and CCK-8 were purchased from Sigma.

***Plasmids, RNA interference and transfection***

Plasmids expressed HA-tagged LPL (pcLPL) and HA-tagged ZHX2 (pcZHX2) were constructed by inserting full-length human LPL and ZHX2 cDNA into the pcDNA3.0 (Invitrogen), respectively. pGL3-LPLp luciferase reporter plasmid was constructed by inserting the promoter of LPL gene into pGL3-Basic vector (Promega). A series deletion of LPL promoters was generated by using KOD-Plus-Mutagenesis Kit. All recombinant plasmids were confirmed by DNA sequencing. siRNAs targeting LPL: 5′-GCAGGAAGTCTGACCAATATT-3′, was synthesized chemically (GenePharma). Cells were transfected with the indicated plasmids or siRNA using Lipofectamine™ 2000 (Thermo Fisher Scientific) according to the manufacturer’s protocol.

***qRT-PCR and western blot***

Total RNA was extracted using TRIzol reagent (TaKaRa), and 1 μg RNA was used to synthesize cDNA using the RevertAid First Strand cDNA Synthesis Kit (Thermo Fisher Scientific). qPCR analysis of gene expression was performed by SYBR Premix Ex Taq™ (TakaRa) according to the manufacturer’s protocol. Relative gene expression was normalized to the internal control GAPDH. Western blot analysis was performed as described previously ^1^.

***Flow cytometry and fluorescent microscopy***

Cells were stained with BODIPY (2μM) for 15min. Dil-VLDL (5μg/ml) was added to the medium and incubated with cells for 2h. Then the treated cells were collected to perform flow cytometry. A minimum of 20,000 events were used to analyze data as mean of fluorescence intensity. Cells were fixed in 4% PFA for 30min at room temperature, and stained the nucleus with DAPI. Then the slides were imaged immediately by fluorescent microscope.

***Luciferase assay***

Pre-cultured cells were transfected with a combination of pGL-LPLp, pRL-TK, and ZHX2 expressed plasmids or ZHX2 siRNA. After incubation at 37°C for 48h, the transfected cells were collected to analyze firefly luciferase activity using the dual-luciferase reporter assay system (Promega) according to the manufacturer’s protocol and normalized to renilla luciferase activity.

***Chromatin immunoprecipitation assays***

ChIP assays were performed according to previous protocol in our lab ^2^. Briefly, Bel7402-ZHX2-Teton cells were cultured in the medium with or without Dox, then fixed cells were sonicated to shear DNA to 200~1000bp and immunoprecipitated using anti-HA antibody (ab9110, abacam) or control IgG (sc-2027, Santa Cruz). qPCR was performed using specific primers targeting the region of LPL promoter.

***In vivo xenograft liver tumor assays***

Male BALB/c mice (6~8 weeks) were housed under specific pathogen-free conditions according to protocols approved by the Shandong University Animal Care Committee. H22 cells xenograft tumors were prepared and plasmids (pcDNA3.0, pcZHX2, pcLPL, pcZHX2+pcLPL) were injected every three days as described before^3^. The tumor volume and tumor weight were estimated.

***NAFLD and NAFLD-HCC model***

NAFLD mice model was induced by feeding with MCD diet or HFD. Briefly, 8-week-old mice were fed with MCD diet (MD12052, Medicinece, Jiangsu, China) for 4 weeks. Four-week-old mice were fed with HFD (MD12032, Medicinece, Jiangsu, China) for 12 weeks. Mice were euthanized at the end of experiments, and liver tissues were collected to make frozen sections or stored in -80℃. Frozen sections were stained with 3% Oil Red O for lipids deposition. The stored tissues were used to detect ZHX2 expression by Western blot.

NAFLD-HCC was induced in male mice by a single subcutaneous injection of 200μg STZ (Sigma) 2 days after birth. At week 4, mice were injected with AAV viruses expressing ZHX2 or LPL (5x10^11^ PFU) and then fed with HFD^4^. At mouth 5, tumor nodes in mice livers were analyzed by magnetic resource scan. Then the mice were euthanized, livers were collected to calculate numbers of tumor nodes, and make frozen sections and paraffin embedded sections. Hematoxylin and eosin (H&E) staining was used to define the steatosis of liver tissues.

**Reference:**

1. Qiu Y, Shen Y, Li X, Liu Q, Ma Z. Polyclonal antibody to porcine p53 protein: a new tool for studying the p53 pathway in a porcine model. *Biochemical and biophysical research communications* 2008, **377**(1)**:** 151-155.

2. Yue X, Zhang Z, Liang X, Gao L, Zhang X, Zhao D*, et al.* Zinc fingers and homeoboxes 2 inhibits hepatocellular carcinoma cell proliferation and represses expression of Cyclins A and E. *Gastroenterology* 2012, **142**(7)**:** 1559-1570 e1552.

3. Zhang H, Song Y, Yang H, Liu Z, Gao L, Liang X*, et al.* Tumor cell-intrinsic Tim-3 promotes liver cancer via NF-kappaB/IL-6/STAT3 axis. *Oncogene* 2018, **37**(18)**:** 2456-2468.

4. Fujii M, Shibazaki Y, Wakamatsu K, Honda Y, Kawauchi Y, Suzuki K*, et al.* A murine model for non-alcoholic steatohepatitis showing evidence of association between diabetes and hepatocellular carcinoma. *Med Mol Morphol* 2013, **46**(3)**:** 141-152.
